# Supplementary material for: A PRISMA systematic review of adolescent gender dysphoria literature: 3) treatment
Source: PLOS Glob Public Health. 2023 Aug 8;3(8):e0001478. doi: 10.1371/journal.pgph.0001478 (PMC10409298; doi:10.1371/journal.pgph.0001478)
Supplement: Attachment — Submitted filename: Response to reviewers.docx [file pgph.0001478.s001.docx]

**THE SAHLGRENSKA ACADEMY**

**Institute of Neuroscience and Physiology**

**Gillberg Neuropsychiatry Centre**

**Kungsgatan 12**

**411 19 Göteborg**

Dear Vanessa Carels

Re: A PRISMA systematic review of adolescent gender dysphoria literature: 3) treatment

We very much appreciate the constructive comments from reviewers on our manuscript. We have addressed each of their points in turn in the table below. In addition to the requested changes, we have implemented a few edits for typographical errors and removal of redundant text. All documents have been uploaded in the appropriate format. We hope this will allow our paper to be published in the near future.

|  | **Reviewer comment** | **Authors’ response** |
| --- | --- | --- |
| Editor | Please review your reference list to ensure that it is complete and correct. If you have cited papers that have been retracted, please include the rationale for doing so in the manuscript text, or remove these references and replace them with relevant current references. Any changes to the reference list should be mentioned in the rebuttal letter that accompanies your revised manuscript. If you need to cite a retracted article, indicate the article’s retracted status in the References list and also include a citation and full reference for the retraction notice. | We have reviewed the reference list and, using both the original online journal record and the Retraction Watch database (<https://retractionwatch.com/>), can find no evidence for any of the papers having been retracted. If the editor is aware of any specific publications which have been retracted since our submission, we would be grateful if you could let us know as we will be happy to make edits accordingly.  We note two cited papers have since had errata published (Arnoldussen et al, 2019 & Bustos et al, 2021), neither of which made any material difference to the outcomes of the papers.  We therefore have not currently made any changes to the reference list. |
|  | Please upload a copy of Figure 2 which you refer to in your text on page 9. Or, if the figure is no longer to be included as part of the submission please remove all reference to it within the text. | Figure 2 was included in the main manuscript, on p30. However, I realise this should have been included as a separate figure file. This has now been uploaded, labelled ‘Fig2.tif’. |
| 1.1 | 1&2. the manuscript has gone through rigorous exercise of systematic review though no statistics was applied to this review. Authors have provided an explanation for not including it. They have used a qualitative approach for this review. 3. Yes the authors have made the document available 4. There are grammatical and writing issues in this paper that I have mentioned in my review. Overall, standard of writing is acceptable but need improvement. | Thank you for your positive comments, and constructive input. These points are addressed below. |
| 1.2 | (a) Authors can take into consideration the consistency in citing the papers in this review. Some places, papers were mentioned but no in-text citation was provided, also authors of the papers were mentioned with no in -text citations. Authors can choose any one of the formats and keep it consistent for ease of reading. (Just my suggestion, Editors can review this)   For example:  Line 276: Four papers (Becker-Hebly et al, 2020; Costa et al, 2015; Kuper et al, 2020; Russell et al, 2020) reported…  Line 297: Three papers^34 39 40^ analysed the effects of GD treatment on …  Line 322-23: Stoffers et al^40^ found that other lipid parameters were unaffected, whereas Klaver et al (2020)^34^ noted a significant increase in total cholesterol, LDL and triglycerides.  Line 352-53: Four papers analysed changes in liver enzymes. Jensen et al^48^ simply noted whether there was any change but did not provide values nor which enzymes were measured. Two studies^36 40^ analysed the effects of testosterone and found differing increases in enzymes.  Line 376-77: Klaver (2020) noted a higher prevalence of obesity in their follow-up at 22 years old in both NM and NF participants. Tack et al (2016)^36^ also noted significant weight gain in NF (but not in NM in their 2017 paper^37^). | Thank you – we can see this inconsistency is jarring for the reader. We have endeavoured to ensure that the reader can always readily see which study we are referring to in results. We would also like to avoid unnecessary repeated citation. We have made corrections wherever we feel the specific papers referred to have not been obvious. Where no in-text citation has been provided, this is where the paper in question is then immediately discussed (and cited) in the following sentence, or because the relevant data table is referred to in the paragraph and the papers can be seen there. |
|  | (b) Authors can consider using short sentences, some were long:   For example: Line 454-57, Line 470-72, Line 517-20 | We believe the first 2 examples given are of appropriate length for an academic publication, with use of a colon in each breaking down the sentence. We have split the third example into 2 sentences. |
|  | (c) Clarity in some passages:   Line 276-82: percentage of patients are reported (7.5% & 28%) but no total number provided for these samples.  Line 388: BMD in relation to GnRHa treatment was examined in three papers. What are those three papers? No reference was provided in this sentence.  Line 413-14: Three of the studies used the Children’s Global Assessment Scale (CGAS^52^), …  What are those three studies? | Line 276-82 – We have added in the N in this section. We also noticed an error in one of the sentences, which has been corrected.  Line 388 – we have added the relevant paper citations.  Line 413-14 – we have added the relevant paper citations to this sentence. |
|  | (d) Grammatical issues: the authors can improve the writing standards. There are long sentences and the reporting of the studies as well as citation are not consistent. Instead of mentioning – “one paper” or “other paper”, authors need to specifically cite the works or the authors. | The entire paper has been re-read for grammatical standard, and some edits made. We appreciate the reference to ‘one paper’, etc, is not always ideal. We have endeavoured to amend this where we can, although the nature of the paper means we have felt it necessary to apply this style to avoid constant repetition of citations. We have used this style in the previous two papers in this series. |
| 2.0 | Really enjoyed reading this paper; it is a well balanced and well written review; it is a bit unfortunate that from the included data to draw definitive conclusions regarding the safety of treatment; I am interested in knowing the difference is GD rate between DSD population and non DSD population; is it possible to include that in the results/ discussion? | Thank you for your kind feedback. We agree that it is unfortunate that we cannot draw definitive conclusions.  We are unable to provide any data on DSD as papers including DSD samples were excluded at the screening stage. This would need to be the focus of a new systematic review. |

We look forward to hearing from you.

Yours sincerely

Lucy Thompson (on behalf of the authors)
